# Using the Community Readiness Model to Select Communities for a Community-Wide Obesity Prevention Intervention

**Published:** 2011-10-15

**Authors:** Sarah Sliwa, Jeanne P. Goldberg, Valerie Clark, Bridgid Junot, Elizabeth Nahar, Miriam E. Nelson, Alison Tovar, Christina D. Economos, Jessica Collins, Ruth Edwards, Raymond R. Hyatt

**Affiliations:** New Balance Doctoral Fellow in Childhood Nutrition, Friedman School of Nutrition Science and Policy, Tufts University; John Hancock Research Center on Physical Activity, Nutrition, and Obesity Prevention, Friedman School of Nutrition Science and Policy, Tufts University, Boston, Massachusetts; John Hancock Research Center on Physical Activity, Nutrition, and Obesity Prevention, Friedman School of Nutrition Science and Policy, Tufts University, Boston, Massachusetts; John Hancock Research Center on Physical Activity, Nutrition, and Obesity Prevention, Friedman School of Nutrition Science and Policy, Tufts University, Boston, Massachusetts; John Hancock Research Center on Physical Activity, Nutrition, and Obesity Prevention, Friedman School of Nutrition Science and Policy, Tufts University, Boston, Massachusetts; John Hancock Research Center on Physical Activity, Nutrition, and Obesity Prevention, Friedman School of Nutrition Science and Policy, Tufts University, Boston, Massachusetts; John Hancock Research Center on Physical Activity, Nutrition, and Obesity Prevention, Friedman School of Nutrition Science and Policy, Tufts University, Boston, Massachusetts; John Hancock Research Center on Physical Activity, Nutrition, and Obesity Prevention, Friedman School of Nutrition Science and Policy, Tufts University, Boston, Massachusetts; Partners for a Healthier Community, Springfield, Massachusetts; FlashStone Research and Consulting LLC, Stilwell, Kansas; Public Health and Family Medicine, Tufts University School of Medicine, Boston, Massachusetts

## Abstract

To build on a growing interest in community-based obesity prevention programs, methods are needed for matching intervention strategies to local needs and assets. We used the Community Readiness Model (CRM), a structured interview guide and scoring system, to assess community readiness to act on childhood obesity prevention, furthering a replication study of a successful intervention.

Using the CRM protocol, we conducted interviews with 4 stakeholders in each of 10 communities of similar size, socioeconomic status, and perceived readiness to implement a community-wide obesity prevention intervention. Communities were in California, Florida, Illinois, Massachusetts, New York, North Carolina, Pennsylvania, and Tennessee. The 4 stakeholders were the mayor or city manager, the school superintendent, the school food service director, and a community coalition representative. Interviews were recorded and professionally transcribed. Pairs of trained reviewers scored the transcriptions according to CRM protocol. The CRM assesses 9 stages of readiness for 6 dimensions: existing community efforts to prevent childhood obesity, community knowledge about the efforts, leadership, community climate, knowledge about the issue, and resources. We calculated an overall readiness score for each community from the dimension scores.

Overall readiness scores ranged from 2.97 to 5.36 on the 9-point scale. The mean readiness score, 4.28 (SD, 0.68), corresponds with a "preplanning" level of readiness. Of the 6 dimensions, community climate varied the least (mean score, 3.11; SD, 0.64); leadership varied the most (mean score, 4.79; SD, 1.13).

The CRM quantified a subjective concept, allowing for comparison among 10 communities. Dimension scores and qualitative data from interviews helped in the selection of 6 communities for a replication study.

## Community Strategies for Reducing Childhood Obesity

To address an issue as complex as childhood obesity, interventionists and practitioners need to implement strategies at numerous levels (1). Approaches that solely address individual behavior have not proved sufficient to prevent obesity at population levels (2). Multicomponent school-based interventions have yielded mixed results (3-6), leaving some to conclude that researchers need to look beyond the school (7). In a meta-analysis of obesity interventions among racial/ethnic minority children, interventions with 3 or more components appeared to be more effective than interventions targeting fewer areas, affirming the value of a multipronged approach (8). The recognition of social, cultural, and environmental factors influencing obesity has motivated a shift to community strategies, on the assumption that change at this level will encourage and sustain individual-level behavior change (9,10).

Experts have identified integrated community-based collaborations among promising approaches to childhood obesity prevention (7,10-16). Government agencies, research institutes, foundations, professional associations, and the private sector are expanding their support for the implementation of these approaches (10-17). Recently, an expert panel identified the need to "understand how local communities can be mobilized to initiate policy change" as a priority for addressing research gaps related to obesity, diet, and physical activity (18). Not all communities may be ready to mobilize around these issues (19).

The realization of a community-based approach depends on an engaged and active base of citizens (20). A lack of broad community participation can hamper implementation and limit the effectiveness of well-intended prevention programs (21,22). As funding opportunities for community-based obesity research continue to grow, so too must researchers' ability to assess community readiness and to tailor strategies accordingly.

Few evaluated multilevel community interventions to prevent childhood obesity exist (5), but those described in peer-reviewed journals have shown promising results. Be Active Eat Well, set in rural Australia, and Shape Up Somerville: Eat Smart, Play Hard, set in a densely populated city in the United States, have demonstrated that community capacity building coupled with changes at multiple levels (eg, parents, schools, communities) can lead to significant reductions in undesirable weight gain among children (23,24).

Before a proven intervention model can be implemented elsewhere, the target community must demonstrate willingness to change and the collective ability to address the chosen issue. Here we demonstrate the application of an existing method to assess community readiness and compare multiple communities in a competitive process to select those sufficiently ready to replicate a successful community-based obesity prevention model.

## Community Readiness to Change

Community readiness can be understood as the observable and psychological characteristics of a community that influence its ability to initiate change (25,26), including, but not limited to, organizational resources and the capacity and attitudes of the community (27). Community readiness has been associated with the perceived effectiveness of a community coalition, which may play a critical role in the implementation of a community-wide initiative (27,28).

Just as individuals progress through stages of change, so do communities. Researchers at the Tri-Ethnic Research Center for Prevention Research at Colorado State University (www.triethniccenter.colostate.edu) developed the Community Readiness Model (CRM) to quantify community readiness to address a specific issue (29). The CRM draws from the transtheoretical model of individual stages of change and theories of community-level processes and social action to measure progress in group change (19,28). Community change demands psychological readiness for change. Problems must be defined, and mechanisms for making decisions, taking action, and sustaining efforts across multiple levels must be in place (28). The CRM stages of "preplanning" and "preparation," which roughly parallel "preparation" in the transtheoretical model, are the earliest stages in which a community would likely be able to implement the intervention within the project time frame. Public health interventions have addressed various issues using the CRM (eg, substance abuse [30], bike helmet use [31], obesity prevention [32,33]).

## Children in Balance Research Initiative

Children in Balance (www.childreninbalance.com) is a research initiative for childhood obesity prevention at Tufts University. The Children in Balance research team initiated Shape Up Somerville (2002-2005), a partnership of 3 culturally diverse urban communities (and 2 control communities) to evaluate whether a multilevel intervention could prevent a rise in body mass index *z *score (BMI-z) in young children who resided in Somerville, Massachusetts (www.nutrition.tufts.edu/index.php?q=research/shapeup-somerville). The intervention aimed to bring early-elementary schoolchildren into energy balance through increased access and availability of healthy food and physical activity options throughout the entire day. Components included in-school and after-school curricula, a school food service intervention, capacity-building activities (eg, training) for school and city personnel, a communications campaign, a restaurant initiative, community events, and parent outreach (24,34,35).

In 2007, Children in Balance announced a request for applications for The Balance Project: Bringing Healthy Eating and Active Living to Children's Environments, a 2-year multilevel randomized, controlled replication of the community-based study. The study design included 3 intervention communities that would receive funding and technical support to implement intervention components and 3 control communities that would receive an annual stipend for serving as controls and materials and training following completion of the intervention. The Tufts University institutional review board approved this study.

The replication study was designed to further the goals of the original intervention; however, communities in the replication trial would have the flexibility to develop and implement their own strategies, policies, and initiatives to meet these goals. The study also offered an innovation grant — outside of intervention funding — to support an initiative of the community's design. The study team would provide technical assistance, workshops, and training sessions to help develop community capacity.

## The Application Process

The main components of the application process were a letter of intent with a deadline of November 15, 2007, a full application (if invited by the review committee) with a deadline of December 17, 2007, and CRM interviews in January 2008.

**Table:** Box 1. Community Eligibility Criteria for Children in Balance Replication Trial

**Criterion**	**Description**
Urban	Must be an incorporated, urban city (US Census definition of *urban*).
Diversity	Racially, ethnically, and economically diverse. (City can make case for diversity. Benchmark used was 60% of school children were eligible for free or reduced-priced lunch.)
Size	Population of 50,000 to 125,000.
Leadership	Independent government structure including an elected mayor.
Coalition	Community-based coalition working on issues of or related to childhood obesity to demonstrate capacity to mobilize around the issue.
Readiness	Must demonstrate an appropriate level of readiness to act, while having not yet engaged in any major prior or current school-wide or community-wide childhood obesity intervention.
Independent food service	Must have a school district with a self-operating food service department. Food service cannot be outsourced.
Professional development	Demonstrated willingness to set aside 1 professional development day per year for teachers, food service staff, and nurses.
Curriculum implementation	Be willing to implement a nutrition and physical activity curriculum at least once per week during the school day (for grades 1 through 3).
Leadership support	Letter of support from the school district superintendent.
Sustainability	Applicant must identify how it would contribute $100,000 in cash or in-kind during 2 years of the project and identify programs that the community or coalition has been successful in piloting and sustaining.

**Table:** Box 2. 9-Point Readiness Scale for Community Readiness Model^a^

Score	Stage	Description
1	No awareness	Issue is not generally recognized by the community or leaders as a problem (or it may truly not be an issue).
2	Denial/resistance	At least some community members recognize that it is a concern, but there is little recognition that it might be occurring locally.
3	Vague awareness	Most feel that there is a local concern, but there is no immediate motivation to do anything about it.
4	Preplanning	There is clear recognition that something must be done, and there may even be a group addressing it. However, efforts are not focused or detailed.
5	Preparation	Active leaders begin planning in earnest. Community offers modest support of efforts.
6	Initiation	Enough information is available to justify efforts. Activities are under way.
7	Stabilization	Activities are supported by administrators or community decision makers. Staff are trained and experienced.
8	Confirmation/expansion	Efforts are in place. Community members feel comfortable using services, and they support expansions. Local data are regularly obtained.
9	High level of community ownership	Detailed and sophisticated knowledge exists about prevalence, causes, and consequences. Effective evaluation guides new directions. Model is applied to other issues.

a Source: Plested et al (29).

The request for applications was publicized on 33 professional listserves and distribution lists, posted on the Children in Balance website, and disseminated through a direct-mail campaign to mayors and school superintendents in 426 urban communities whose populations met the criteria listed in Box 1. The study team held an optional webinar and conference call for interested applicants 2 weeks before the letter of intent deadline. Attendees received information about the study background, design, theory, eligibility criteria, and application time line and asked questions. Potential applicants also e-mailed study staff with questions.

Sixty-seven participants from 54 communities in 26 states participated in the webinar. The study team reviewed 30 letters of intent and invited all 26 eligible communities to submit full applications. Twenty-two communities from 17 states submitted full applications. A team of 12 reviewers, 6 internal (staff and faculty) and 6 external (from foundations, academia, and government), independently reviewed and scored the 22 applications, using criteria and a scorecard developed by staff. The team ranked communities by average total score to identify the 10 highest-scoring communities.

Through the competitive grant-application process, the review team sought to identify communities that did not have comprehensive obesity prevention programming but demonstrated existing efforts, leadership, and collaboration as evidence of their potential to implement intervention components.

## Using the CRM Interview Protocol to Assess and Select Finalist Communities

For the next step, the study team used the CRM protocol (29) to select 6 finalist communities from the top 10. Two team members attended a CRM training session at the Tri-Ethnic Center in the fall of 2007 and then trained 4 team members to conduct and score the semistructured interviews by using the Tri-Ethnic Center's protocol (www.triethniccenter.colostate.edu/CRhandbookcopy.htm) (29). This protocol uses a 9-point readiness scale (Box 2) to evaluate 6 dimensions: community efforts to address the issue, community knowledge about the efforts, leadership, community climate (prevailing community attitude), community knowledge about the issue, and resources. The interview protocol includes 36 semistructured questions; 21 are "anchored" questions that directly address at least 1 of the 6 dimensions and are required for assessment (29). The 15 nonanchored questions are optional and can be modified according to researchers' needs. At least 4 to 6 people in each community should be interviewed to assess a community's readiness (29).

The study team modified the CRM interview script to focus on childhood obesity. The study team substituted 1 anchored question with a nonanchored question. The final script (Appendix) had 23 questions, including 20 anchored questions. The 3 additional questions addressed existing policies, practices, or laws related to obesity and the identification of community leaders on the issue. These questions were relevant because of the project's objectives to create policy change and sustain leadership support.

Interviewers conducted the interviews during 2 weeks in January 2008. In each community, interviewers contacted the mayor or city manager, school district superintendent, school food service director, and a representative from the community coalition that submitted the application. Four people were interviewed in each community, for a total of 40 interviews. Interviewers used voice-over Internet protocol telephones and digitally recorded the calls with permission from interviewees. Interviews lasted approximately 30 minutes. A transcription agency transcribed the audio files.

Paired scorers reviewed the transcripts and assigned scores (Box 2) for each of the 6 dimensions independently. Scores are assigned on a scale of 1 to 9, in 0.25 increments (ie, 1.00, 1.25, 1.50, etc.). Each pair met to discuss the dimension scores and achieve consensus. According to CRM protocol, reviewers averaged the consensus scores for each dimension and rounded them down to the nearest integer to determine the overall CRM score. No study member scored transcripts from communities in which they had conducted interviews.

## Community Readiness Model Scores

The mean overall CRM score for the 10 communities assessed was 4.28 (SD, 0.68), corresponding with the preplanning stage of readiness. Overall scores ranged from 2.97 to 5.36. Among the scores for the 6 dimensions (averaged for the 10 communities), the lowest were for community climate (mean, 3.11; SD, 0.64), knowledge about the issue (mean, 3.61; SD, 0.80), and knowledge of efforts on the issue (mean, 3.80; SD, 0.55) — all corresponding with the vague awareness stage. The leadership score (mean, 4.79; SD, 1.13) varied the most, corresponding with preplanning. The score for resources related to the issue (mean, 4.76; SD, 0.68) also corresponded with preplanning. The score for existing community efforts (mean 5.64; SD, 0.96) was the highest and corresponded with the preparation stage, indicating that these communities were likely launching and planning efforts with modest support from the community.

The review committee reconvened to discuss the CRM scores, CRM interview results, and other application components, including community demographics and coalition formation. The committee was particularly interested in the scores for leadership and community knowledge of the efforts because they reflect the support of key community decision makers and the level of awareness among citizens and leaders. The committee then ranked the communities to identify 6 finalists and 2 alternates.

## Site Visits to 6 Finalist Communities

The study team visited the 6 finalist communities to confirm eligibility and application information. Each visit included an observation of a school lunch, a community tour to understand local infrastructure, and semistructured interviews with community leaders in child health, which included some CRM interview participants. During these site visits, the study team interviewed an average of 11 people (range, 6-20) per community; some sites organized 15 to 20 coalition members for a group interview. The site-visit interviews addressed resources, local leadership, current initiatives, and community motivation. Questions for the superintendent and food service director focused on willingness and ability to promote physical activity, healthy eating, and school wellness practices. Study team members asked community leaders who were not part of the CRM interviews 8 questions from the CRM script. These interviews were not scored. As a result of the site visits, 1 community was deemed ineligible because the school district outsourced its food service. The study team visited and confirmed the eligibility of an alternate community.

## Interpretation of CRM Scores

The overall CRM scores demonstrate that the applicant communities ranged from vague awareness to preparation stages of change (scores of 3-5); these scores matched the review committee's interest in identifying communities that were eager to address childhood obesity but did not already have comprehensive efforts in place. The letter of intent asked applicants to describe previous efforts that had been sustained. In the CRM stages, evidence of collaboration on the issue — a desired attribute — would correspond more closely with an overall score of preplanning (score of 4) or preparation (score of 5).

The low score (vague awareness) for community climate may indicate limited community empowerment (29). Similarly, the vague awareness of knowledge about obesity suggests limited recognition of childhood obesity as a local problem. In contrast, the mean score for existing community efforts was the highest of the dimensions. The difference between these scores indicates that efforts existed and communities *could* have been informed of them; the limited awareness suggests a communication gap between the community and the people involved in obesity prevention efforts.

The variability among the leadership scores indicated differences in community leaders' prioritization of childhood obesity prevention among competing issues. Leadership support is necessary for ensuring the sustainability of a community-wide project; for this reason, low leadership scores (≤3.9) were a red flag to review committee members.

Communities with a high overall score (eg, a score of 6, corresponding with the initiation stage of readiness) may already have sufficient motivation and momentum to initiate and sustain intervention components on their own (19). Communities with a low overall score (eg, a score of 2, corresponding with denial/resistance) would need to dedicate significant efforts to raising awareness and building relationships in advance of implementing any intervention components. For this reason, the review committee removed from consideration a community with an overall score of 2.97.

The qualitative information gained from the interviews enhanced the study team's understanding of the applicant communities and in 1 case, led to the elimination of an applicant. This finalist demonstrated a robust coalition, innovative programming, and a multicultural population in its written application. During the CRM interviews, however, it emerged that the community was deeply divided along ethnic lines, which would likely impede implementation of a community-wide, collaborative program.

The CRM transcripts provided descriptive information about existing programs, policies, challenges, and resources that can be leveraged for the intervention (32). Identifying local experts and seeking their opinion through interviews had additional value as an entry point to securing support from community leaders.

The 4 community members selected for the interviews were in leadership positions whose support and collaboration seemed integral to achieving the study objectives. The community, however, may not have seen these people in the same light. By preselecting interview participants on the basis of predetermined criteria, the study team may have overlooked other important community perspectives.

Because the interviews took place within a competitive application process, they may reflect social desirability bias. Respondents may have overstated community activities and commitment to childhood obesity prevention in hope of securing funding. Additionally, the transcript scoring process demands interpretive discretion, which the consensus process aims to attenuate.

The study team did not conduct reliability tests for the modified protocol. Because the only change was the substitution of a question addressing community climate, the change is not expected to have compromised accuracy of either the dimension or overall scores; of the 6 dimensions, community climate is seen as the least concrete and is often inferred from answers to anchored questions addressing other dimensions. The study team trained the scoring pairs to identify statements corresponding to each dimension throughout the interview transcripts.

Community readiness is a subjective construct. The CRM scoring system assigns numerical values to ease comparison; however, the data are fundamentally qualitative (26). The CRM captures a snapshot of a community during the interview period; a community, however, is constantly changing and readiness can be in flux. A crisis, or a change in leadership, may redirect community motivation and resources. Establishing the validity of a community readiness measure is challenging in the absence of a true readiness value that could be captured through an objective protocol.

A previous application of the CRM to obesity prevention used the overall readiness score to design an intervention that would be appropriate for a specific community and its level of readiness (32). That approach was similar to previous uses of the CRM for strategic planning for public health issues. Researchers have also applied the CRM as a pre–post measurement for a randomized community intervention (19,36); the CRM score was used to identify communities that would be able to implement an existing intervention model. To our knowledge, this is the first application of the CRM to compare readiness within a competitive request for applications addressing the issue of childhood obesity prevention.

The CRM protocol enabled the study team to gather information remotely about community capacity for an issue of interest. This ability reduced the number of site visits needed, thereby lowering travel costs and overall costs in the review process.

The stage model of the CRM is sufficiently concrete to be useful to researchers, consultants, and evaluators who wish to provide feedback to communities (25,28). Finding comparison sites for community-level interventions is difficult (37). The CRM scores provided an additional level of comparison among 10 communities of similar size, diversity, socioeconomic status, and perceived ability to implement a 2-year community-based obesity prevention program. The ability to evaluate readiness is central to determining whether a community can successfully execute a given intervention and to identifying a starting place for researchers and practitioners who are designing programs or interventions (29,38). Without this information, programs risk over- or underestimating what communities are capable of implementing (39), making for an inefficient use of resources.

## Appendix A. Website Survey Abstraction Tool^a^

**Table:**

Community Efforts and Community Knowledge of Efforts

Using a scale from 1 to 10, how much of a concern is childhood obesity in your community (with 1 being "not at all" and 10 being "a very great concern")? Please explain.
Describe current efforts in your community to address childhood obesity.
For how long have these efforts been going on in your community?
Using a scale from 1 to 10, describe how aware people in your community are of these efforts (with 1 being "no awareness" and 10 being "very aware"). Please explain.
What does the community know about these efforts or activities (eg, logistics, goals, participants)?
What are the strengths of these efforts?
What are the weaknesses of these efforts?
*What formal or informal policies, practices, and laws related to childhood obesity are in place in your community, and for how long? Prompt: A *"*formal*"* policy would be an established policy in schools. An informal policy would be an *"*unsaid rule*"* or pattern of behavior.*

**Leadership**

*Who are leaders/community champions specific to the childhood obesity issue in your community?*
Using a scale from 1 to 10, how much of a concern is childhood obesity to the leadership in your community (with 1 being "not at all" and 10 being "of great concern")? Please explain.
How are leaders getting involved in this issue? Prompt: Are they involved in a committee, a task force? How often do they meet?
Would leadership support additional efforts and services? Please explain.

**Community Climate^b^**

*What are community perceptions of childhood overweight?*
What are the primary obstacles to efforts addressing this issue in your community (eg, language, competing interests, structure of the school district)?

**Knowledge About the Issue**

How knowledgeable are community members about childhood obesity? Prompt: Are they familiar with signs, symptoms, effect on family?
What type of information is available in your community regarding childhood obesity?
What local data are available on this issue in your community?
How do people obtain this information in your community?

**Resources for Prevention Efforts**

To whom would an individual affected by childhood obesity turn to first for help in your community? Why?
What is the community's and/or local business's attitude about supporting efforts to address this issue, with people volunteering time, making financial donations, and/or providing space?
Are you aware of any proposals or action plans that have been submitted for funding that address childhood obesity in your community? If yes, please explain.
Do you know if there is any evaluation of efforts that are in place to address this issue? If yes, on a scale of 1 to 10, how sophisticated is the evaluation effort (with 1 being "not at all" and 10 being "very sophisticated")?
How are the evaluation results being used (ie, to make changes in programs)?

a Nonanchored questions are presented in italics. The Community Readiness Model interview protocol (29) includes anchored questions (required) and nonanchored questions (optional).

b The Tri-Ethnic Center for Prevention Research, which developed the interview protocol for the Community Readiness Model, recommends the inclusion of 21 anchored questions (29). The protocol used for this project included 20 anchored questions. In the category Community Climate, a nonanchored question (What are community perceptions of childhood overweight?) was used to replace an anchored question (How does the community support the efforts to address this issue?).
